# Natural sea salt consumption confers protection against hypertension and kidney damage in Dahl salt-sensitive rats

**DOI:** 10.1080/16546628.2017.1264713

**Published:** 2016-12-20

**Authors:** Bog-Hieu Lee, Ae-Ri Yang, Mi Young Kim, Sara McCurdy, William A. Boisvert

**Affiliations:** ^a^ Department of Food and Nutrition, School of Food Science and Technology, Chung-Ang University, Seoul, Korea; ^b^ Center for Cardiovascular Research, University of Hawaii John A. Burns School of Medicine, Honolulu, Hawaii; ^c^ Institute of Fundamental Medicine and Biology, Kazan Federal University, Kazan, Russia

**Keywords:** Salt consumption, blood pressure, hypertensive rat model, glomerulosclerosis, echocardiography

## Abstract

Although sea salts are widely available to consumers nowadays, whether its consumption over refined salt has any real health benefits is largely unknown. This study was conducted to compare hypertension-inducing propensity of natural sea salt (SS) to refined salt (RS) in a well-established animal model of hypertension. Five groups of male Dahl salt-sensitive rats were fed rat chow diet supplemented with various amounts of salt for 15 weeks. The groups were: control (CON, *n* = 10), 4% RS (RS4), 4% SS (SS4), 8% RS (RS8), 8% SS (SS8) (*n* = 12 for each group). After 15 weeks, both SS4 and SS8 groups had significantly lower systolic (SBP) and diastolic blood pressure (DBP) compared to RS4 and RS8 rats, respectively. RS8 rats had markedly higher SBP and DBP compared to all other groups. Echocardiography just prior to sacrifice showed abnormalities in RS4, SS8 and RS8 hearts, while CON and SS4 hearts displayed normal measurements. Plasma renin and aldosterone levels of high salt groups were lower than those of CON, and serum electrolytes were similar amongst all groups. Abnormal kidney pathology and high glomerulosclerosis index scores were seen in RS4 and RS8 rats, but SS4 and SS8 kidneys showed relatively normal morphology similar to CON kidneys. Our findings show that consumption of natural sea salt induces less hypertension compared to refined salt in the Dahl salt-sensitive rat.

## Introduction

Hypertension, a physiological state in which the blood pressure is maintained at a high level for prolonged periods of time, is the most representative disease of cardiovascular diseases. It is well known that uncontrolled hypertension contributes to various cardiovascular and other conditions such as coronary heart disease, stroke, congestive heart failure, peripheral vascular disease and renal insufficiency [1]. As excessive ingestion of salt can lead to high blood pressure, which is related to cardiovascular disease and arterial vascular changes, regulation of dietary salt intake is very important. Being one of the major electrolytes, sodium is essential for regulating blood pressure. In addition, sodium homeostasis is critically important for a number of vital cell functions such as excitability, excitation–contraction coupling, energy metabolism, pH regulation, as well as for cardiac development and growth [2]. On the other hand, excessive sodium intake from food sources in close relations to diet and lifestyle is a strong risk factor for hypertension, since the majority of people with hypertension are particularly sensitive to salt intake. However, other risk factors including obesity, diabetes, insufficient intake of potassium, calcium, and magnesium, lack of physical activity, and chronic alcohol consumption play important roles as well [3].

The essential role of dietary salt in hypertension has been emphasized for many years, first by Ambard & Beaujard [4] in 1904 and later by Blackwood [5], Morris [6], and Dahl et al. [7]. These researchers demonstrated unequivocally that dietary sodium intake is associated with blood pressure. Cutler et al. [8] demonstrated that consuming low sodium diet from at least 1–12 months reduces both systolic blood pressure (SBP) and diastolic blood pressure (DBP). Moreover, Bertino et al. [9] and Blias et al. [10] showed that prolonged intake of low sodium diet can lead to enhanced detection of salty taste at low concentrations, which helps to maintain a low sodium diet. Thus, there is ample evidence that salt reduction through low salt diet is critically important for the prevention and management of hypertension. In addition to the amount of salt intake, the selection of salt source may be important for managing hypertension.

The common sources of salt for consumers can be classified into refined (table) salt, sea salt, flower salt, and processed salt [11]. Sea salt contains trace amounts of natural minerals such as MgSO_4_, CaSO_4_, CaCl_2_, and KCl with slightly lower sodium content compared to refined salt. Although there is growing awareness by the general public of the health benefits of sea salt, there is little information on whether consumption of sea salt can have a direct effect on blood pressure regulation [12]. The purpose of this study was to assess if consuming sea salt may have any beneficial effects on blood pressure and its related physiological indices. This was achieved by using male Dahl salt-sensitive (DSS) rats as a model of hypertension and giving diets with two different concentrations of added sea salt or refined salt for a period of 15 weeks.

## Materials and methods

### Animals

Four-week-old male Dahl salt sensitive rats were purchased from Japan SLC, Inc (Shizuoka, Japan). The rats were fed the experimental diet for 15 weeks after 9 weeks of adaptation period and housed at 21 ± 1°C room temperature with 50–60% humidity, and 12/12 hr light/dark cycle. The rats were randomly assigned to five groups: control diet (CON, *n* = 10), 4% sea salt diet (SS4, *n* = 12), 4% refined salt diet (RS4, *n* = 12), 8% sea salt diet (SS8, *n* = 12), and 8% refined salt diet (RS8, *n* = 12). Food and water were available *ad libitum* throughout the 15 weeks. At the end of the 15 weeks the rats were fasted for 12 hours and then anaesthetized with zolazepam (25 mg/kg BW) plus xylazine (10 mg/kg BW) before being sacrificed. All animal work was approved by the Seoul National University Institutional Animal Care and Use Committees (SNU-120316–2) where the rats were housed.

### Diet preparation

Regular rat chow (Teklad Global 18% Protein Rodent diet (Harlan Teklad, WI)) was used as the control diet. Energy composition of rat chow diet was 58% as carbohydrate, 18% as fat, and 24% as protein. Sea salt was from the Jeonnam Sinan region and the refined salt was from the Hanju region of Korea. Feed was made from the powdered form of the chow using the facilities located at the Korea Food Research Institute. Both salts were added to the rat chow on the basis of weight. General composition analysis and mineral contents of the salts was performed based on CODEX STAN 150–1985 and ICP-AES (Activa, HORIBA Jobin-Yvon, Longjumeau, France), respectively. Based on our analysis, on a weight basis the sea salt contained 85.7% NaCl whereas the refined salt contained 99.9% NaCl. In addition to NaCl, the sea salt contained 1.5 mg/g of calcium, 2.9 mg/g of potassium and 3.9 mg/g of magnesium as well as trace amounts of iron, manganese and zinc. Refined salt did not contain any measurable amounts of any other mineral.

### Body weight and feed intake measurement

Body weight was measured once a week during the same time. Feed intake was estimated twice a week by measuring the feed remaining in the cages after 24 hours. Feed efficiency ratio (FER) was calculated from feed intake divided by body weight gain.

### Measurement of blood pressure

Blood pressure was measured in week 1, 2, 3, 10, 12, 13, 14, 15 by tail cuff method using LE 5002 Storage Pressure Meter (Panlab, Barcelona, Spain). The rats were kept at 32–34°C in temperature-controlled heating chamber (Heater Scanner LE 5650/6, Panlab, SI Barcelona, Spain) for 15 min before the cuff was fitted to the tail for 5 min. Blood pressure was measured five times, and the lowest value was used for the results.

### Echocardiography

Six rats were selected randomly in each group for echocardiographic measurement at the end of the study. The M-mode echocardiography system (Sonoace 9900, Madison, WI) was used for the measurement after fasting the rats for 12 hours and anaesthetizing them with zolazepam and xylazine. The measurements included the following: IVSd (interventricular septal thickness at end-diastole, mm), LVDd (left ventricular end-diastolic dimension, mm), IVSs (interventricular septal thickness at end-systole, mm), LVDs (left ventricular end-systolic dimension, mm), LVPWd (left ventricle posterior wall in diastole, mm), LVPWs (left ventricle posterior wall in systole, mm), LV Vol.d (left ventricular volume in diastole, ml), LV Vol.s (left ventricular volume in systole, ml), ejection fraction (%), stroke volume (ml), Fraction shortness (%), and LV mass (left ventricular mass, g).

### Blood and urine measurements

At sacrifice blood samples were drawn from the abdominal aorta and, plasma samples were prepared by centrifugation at 3,000 rpm for 15 min. All samples were stored at – 20°C until analysis.

Aldosterone and renin activity were measured using radioimmunoassay [13]. Electrolytes (Na^+^, K^+^, Cl^−^, Mg^2+^, Ca^2+^) were measured using ion-selective electrode technique by ADVIA 2400 analyser (Siemens, Inc. Munich, Germany), and osmolality was measured using a vapor pressure osmometer 5600 (Wescor, Inc. Logan, UT, USA). Urinary electrolytes and aldosterone were corrected for creatinine.

### Tissue analysis

After 15 weeks of experiment, heart, kidney, spleen, liver, and testicles were dissected out and weighed. The organs were fixed in 10% neutral buffered formalin solution and 3 mm tissue sections were embedded in paraffin using an automated embedder. The paraffin blocks were cut at 3 um thickness using a microtome and subjected to haematoxylin and eosin (H&E) staining to examine the tissue morphology. The kidney sections were subjected to the standard Masson’s trichrome staining protocol from which glomerulosclerosis index was calculated to quantitate renal injury [12].

### Statistical analysis

Statistical analysis was performed using SPSS for Windows 20.0 (SPSS Inc. Chicago, USA). Either one-way analysis of variance (ANOVA) or Student’s *t*-test was used to determine significant differences amongst the groups. The differences between the means of the groups were assessed using Duncan’s multiple range test. Statistical significance was considered at *p* < 0.05.

## Results

### Body weight, feed intake, FER and salt intake

Body weight, feed intake and FER are presented in Table 1. Initial body weight of SS8 group was significantly higher than the other groups (*p* < 0.05) except for RS8 group. However, the mean final weight of RS8 group was significantly lower than all other groups (*p* < 0.001). Body weight gain of CON, SS4, and RS4 groups did not differ, but that of RS8 group (3.68 ± 0.85 g) was the lowest followed by SS8 group (4.65 ± 0.69 g). Feed intake of CON group was the lowest (20.44 ± 0.23 g) and that of SS8 group the highest (26.31 ± 2.24 g) with the other three groups being similar. Although the food intakes were variable, sodium intake between the RS4 and SS4 and between the RS8 and SS8 rats were similar (Table 1). This was due to larger amount of food consumption by the SS groups despite the RS diet containing a higher salt content than the SS diet. The fact that SS diets were consumed at a higher rate than the RS diets may be an indication that the chow with SS was more palatable to the rats. This also precludes salt consumption as the source of differences seen the parameters that were measured between the RS4 and SS4 groups and between the RS8 and SS8 groups.

**Table 1.** Body weight, feed intake, FER and mineral intakes of rats fed high-salt diets for 15 weeks.

Parameters	CON	RS4	SS4	RS8	SS8
Initial weight (g)	366.80 ± 4.29^a^	369.76 ± 4.34^a^	369.33 ± 3.58^a^	374.16 ± 2.35^ab^	384.16 ± 4.69^b^
Final weight (g)	458.60 ± 5.54^b^	461.1 ± 6.42^b^	455.25 ± 5.30^b^	427.85 ± 2.92^a^	453.93 ± 4.11^b^
Weight gain (g)	6.12 ± 0.16^c^	6.09 ± 0.23^c^	5.73 ± 0.21^c^	3.58 ± 0.25^a^	4.65 ± 0.20^b^
Feed intake (g/day)	20.44 ± 0.23^a^	22.49 ± 0.27^b^	23.02 ± 0.14^b^	23.07 ± 0.09^b^	26.31 ± 0.65^c^
Feed efficiency ratio (%)	0.29 ± 0.01^c^	0.27 ± 0.02^b^	0.24 ± 0.01^b^	0.13 ± 0.02^a^	0.16 ± 0.01^a^
Sodium intake (g)	0.04 ± 0.00^a^	0.35 ± 0.00^c^	0.32 ± 0.00^b^	0.72 ± 0.00^d^	0.73 ± 0.02^d^
Potassium intake (g)	0.122 ± 0.001^a^	0.130 ± 0.001^b^	0.133 ± 0.000^b^	0.127 ± 0.000^ab^	0.146 ± 0.003^c^
Magnesium intake (g)	0.041 ± 0.000^a^	0.043 ± 0.000^b^	0.044 ± 0.000^b^	0.042 ± 0.000^ab^	0.049 ± 0.001^c^

Values are presented as mean±SEM. Values with different superscripts within each row are significantly difference at *p* < 0.05 by Duncan’s multiple range test.

### Blood pressure

**Figure 1.**
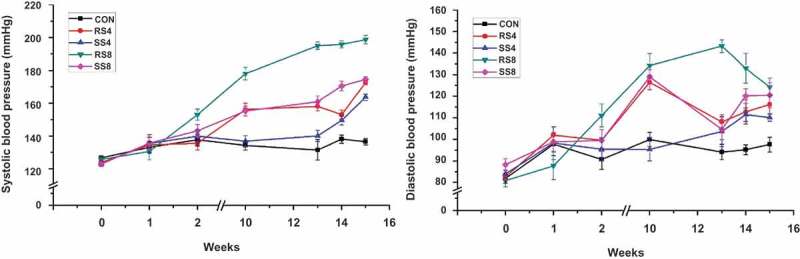
SBP and DBP measurements during the 15-week study period. Values are presented as means ± SEM; an asterisk indicates that a point is significantly different from week 0; *P < 0.05, ***P < 0.001.

Systolic blood pressure (SBP) measurements for the 15-week study period are presented in Figure 1. As expected, SBPs of the salt consumption groups increased steadily, whereas the SBP of the CON group did not change significantly over time. Initial SBP of the experimental groups were similar at approximately 125 mmHg and began to rise slowly 1–2 weeks into the study. After 10 weeks, SBP of RS8 group (178.00 ± 12.04 mmHg) had risen significantly higher in comparison to those of SS4 (136.80 ± 10.41 mmHg), SS8 (155.30 ± 9.35 mmHg) and RS4 (156.17 ± 13.32 mmHg) groups (*p* < 0.001). At week 15, SBP of RS8 group (198.67 ± 8.76 mmHg) was the highest and had increased 72.92 ± 10.09 mmHg in comparison to the initial SBP (*p* < 0.001). SBP of SS8, RS4, and SS4 groups were 174.45 ± 5.52 mmHg, 172.50 ± 4.56 mmHg, and 163.67 ± 5.68 mmHg, respectively. The increase in SBP over the 15 week period for SS8, RS4, and SS4 groups was 51.53 ± 5.97 mmHg, 48.67 ± 7.89 mmHg, 40.59 ± 6.64 mmHg, respectively. For both 4% and 8% groups the mean rise in SBP of the SS rats was significantly lower than in the RS group.

**Figure 2.**
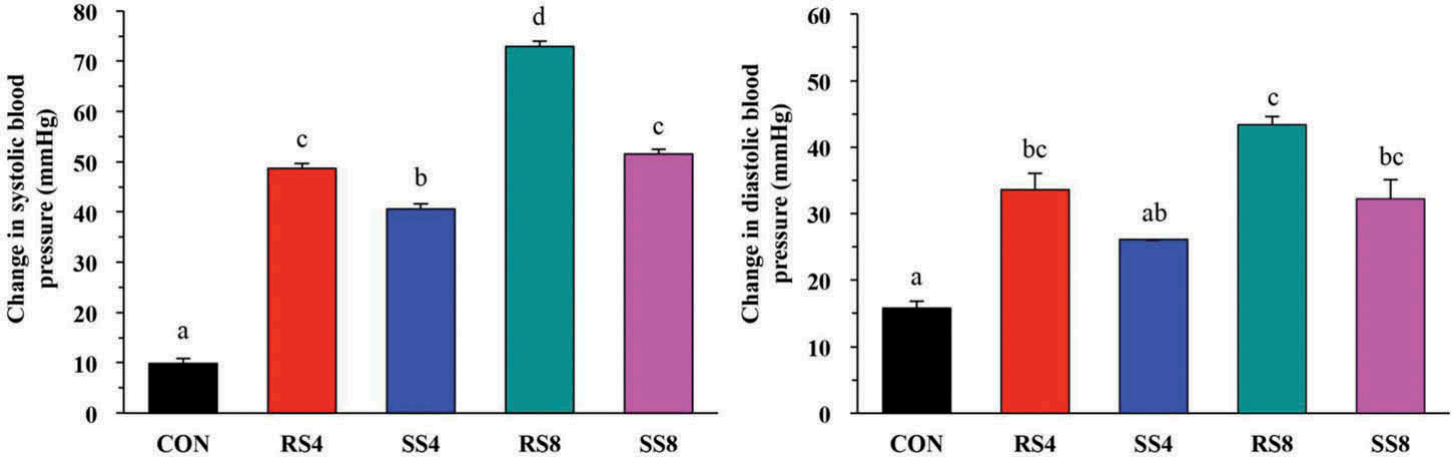
Overall changes in blood pressure of rats fed high salt diets from week 0 to week 15. Data are presented as mean ± SEM. Labeled means without a common letter differ significantly at *P *< 0.05 by Duncan’s multiple range test.

Mean diastolic blood pressure (DBP) of all groups was approximately 85 mmHg and started to increase during the first week. At week 10, DBP had increased sharply, but after week 10, DBP of SS8, RS4, and SS4 groups remained at a similar level. DBP of RS8 group increased the most over the 15 weeks with 43.33 ± 21.18 mmHg and the final DBP was 124.17 ± 14.64 mmHg, the highest among the groups. The rest of the DBP increase over the 15 weeks was as follows: RS4 (33.58 ± 14.92 mmHg), SS8 (32.27 ± 20.69 mmHg), SS4 (26.44 ± 5.53 mmHg), and CON (15.70 ± 19.06 mmHg). Although as with SBP, the DBP change of the SS groups over the 15 weeks for both the 4% and 8% diet groups were lower than for the RS groups (Figure 2), the differences did not reach statistical significance.

### Echocardiography

Echocardiography was performed after 15 weeks of feeding and the M-mode echocardiographic findings are shown in Table 2. Interventricular septal thickness at end-diastolic (IVSd) of SS8 (0.25 ± 0.03 cm) and RS8 (0.25 ± 0.02 cm) was significantly thicker than those of both the CON and SS4 groups (*p* < 0.001). However, IVSd of SS4 group (0.22 ± 0.01 cm) was not different from the CON group although that of RS4 group was thicker than the CON group (*p* < 0.001). Similar trend was seen with interventricular septal thickness at end-systolic (IVSs). There was no significant difference between CON (0.33 ± 0.01 cm) and SS4 (0.34 ± 0.01 cm), but IVSs of RS4 (0.39 ± 0.02 cm), RS8 (0.38 ± 0.03 cm), and SS8 (0.40 ± 0.05 cm) were significantly thicker than the CON and SS8 groups (*p* < 0.001). Left ventricle posterior wall in diastole (LVPWd) and left ventricle posterior wall in systole (LVPWs) of RS4, SS8, and RS8 groups were hypertrophied compared to those of CON and SS4 groups (*p* < 0.05). Left ventricular (LV) mass of three of four high salt diet groups (RS4; 1.68 ± 0.10 mL, SS8; 1.77 ± 0.11 mL, RS8; 1.71 ± 0.10 mL) were higher than that of CON (1.43 ± 0.06 mL) (*p* < 0.001). However, LV mass of SS4 group (1.54 ± 0.08 mL) did not differ from the CON group. Other parameters pertaining to the LV function as well as stroke volume, ejection fraction and fractional shortening were similar amongst the study groups.

**Table 2.** Echocardiography of rats fed high-salt diets for 15 weeks.

	CON(*n* = 6)	RS4(*n* = 6)	SS4(*n* = 6)	RS8(*n* = 6)	SS8(*n* = 6)
IVSd (cm)	0.20 ± 0.01^a^	0.23 ± 0.02^bc^	0.22 ± 0.01^ab^	0.25 ± 0.02^c^	0.25 ± 0.03^c^
IVSs (cm)	0.33 ± 0.01^a^	0.39 ± 0.02^b^	0.34 ± 0.01^a^	0.38 ± 0.03^b^	0.40 ± 0.05^b^
LVDWd (cm)	0.19 ± 0.01^a^	0.21 ± 0.03^ab^	0.19 ± 0.01^a^	0.22 ± 0.03^b^	0.23 ± 0.03^b^
LVPWs (cm)	0.31 ± 0.01^a^	0.36 ± 0.04^abc^	0.32 ± 0.03^ab^	0.37 ± 0.03^c^	0.37 ± 0.06^bc^
LV Mass (g)	1.43 ± 0.06^a^	1.68 ± 0.10^b^	1.54 ± 0.08^a^	1.71 ± 0.10^b^	1.77 ± 0.11^b^

Values are presented as mean±SEM. Values with different superscripts within each row are significantly difference at p < 0.05 by Duncan’s multiple range test.

**IVSd**, interventricular septal thickness at end-diastole; **LVDd**, left ventricular end-diastolic dimension; **IVSs**, interventricular septal thickness at end-systole; **LVDs**, left ventricular dimension in diastole; **LVPWd**, left ventricle posterior wall in diastole; **LVPWs**, left ventricle posterior wall in systole; **LV mass**, left ventricular mass.

### Organ weights

Organ weights at sacrifice are shown in Table 3. Heart weight of CON group (1.65 ± 0.12 g) was significantly lower than those of all other experimental groups (SS4; 1.85 ± 0.23 g, RS4; 1.98 ± 0.17 g, SS8; 1.90 ± 0.14 g, RS8; 1.91 ± 0.11 g) (*p* < 0.001). Kidney weights showed similar tendency with the kidney weight of the CON group (2.88 ± 0.19 g) being significantly lower than those of all of the high salt diet groups (SS4; 3.21 ± 0.16 g, RS4; 3.36 ± 0.1 g, SS8; 3.46 ± 0.14 g, RS8; 3.60 ± 0.23 g) (*p* < 0.001). Liver, spleen, and epidydimal weights were not different amongst the groups.

**Table 3.** Organ weight of rats fed high-salt diets for 15 weeks.

	CON	RS4	SS4	RS8	SS8
Heart	1.65 ± 0.04^a^	1.98 ± 0.05^b^	1.85 ± 0.07^b^	1.91 ± 0.03^b^	1.90 ± 0.04^b^
Kidney	2.88 ± 0.06^a^	3.36 ± 0.03^bc^	3.21 ± 0.05^b^	3.60 ± 0.07^d^	3.46 ± 0.04 ^cd^
Liver	14.54 ± 0.66	14.98 ± 0.65	14.41 ± 0.65	14.70 ± 0.78	14.39 ± 0.57
Spleen	0.85 ± 0.04	0.86 ± 0.03	0.82 ± 0.03	0.89 ± 0.03	0.83 ± 0.04
Testicle	4.16 ± 0.08	4.08 ± 0.14	4.17 ± 0.05	4.03 ± 0.05	4.25 ± 0.08

Values are presented as mean ± SEM. Values with different superscripts within each row are significantly difference at *p* < 0.05 by Duncan’s multiple range test.

### Histology of the kidney

**Figure 3.**
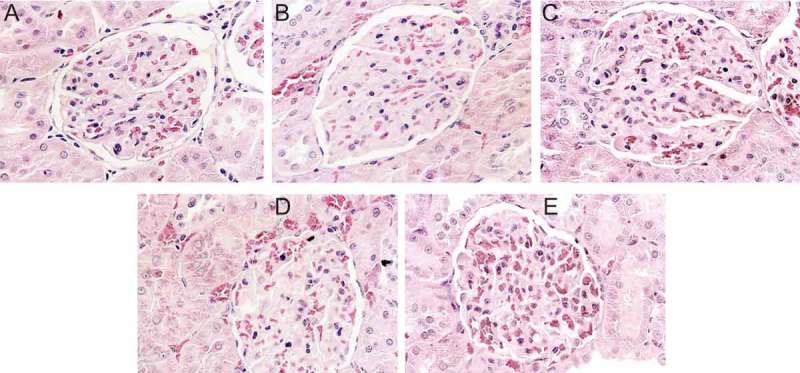
Effect of high-salt diet on kidney histopathology (H & E stain, 400× magnification). (a) CON, no histopathologic lesions are seen. (b) RS4, hyaline matrix is increased in glomerular tufts. Eosinophilic homogenenous hyaline droplets (arrows) are seen in the cytoplasm of proximal convoluted epithelium. (c) RS8, numerous eosinophilic homogeneous hyaline droplets (arrows) are filled with cytoplasm of proximal convoluted epithelial cells. Pale eosinophilic hyaline matrix is increased in glomerular tufts. (d) SS4 and (e) SS8, only scant eosinophilic homogeneous hyaline droplets (arrows) are observed in the cytoplasm of proximal convoluted epithelial cells. No histopathological lesions are seen in the glomerular tufts.

To check for any irregularity of the kidney structure that may have been caused by high salt consumption and salt type, kidney sections were made and stained with haematoxylin and eosin. As shown in Figure 3, whereas the CON group (A) maintained normal structure in the kidneys with intact glomerulus and tubules, mild focal segmental glomerulosclerosis was frequently observed in rats from the RS4 (B) and RS8 (C) groups. These lesions are characterized by sclerosis of some, but not all, glomeruli. In the affected glomeruli, only a portion of the capillary tufts was involved. In addition, numerous hyaline droplets were present as round, eosinophilic droplets within the cytoplasm of the proximal convoluted tubular epithelial cells in the kidneys of RS4 and RS8 rats. The lumen of tubular cells was filled with proteinous materials, indicating renal disease associated with protein loss in the urine (proteinuria). On the other hand, SS4 (D) and SS8 (E) rat kidney sections displayed much less structural disruption in which much of the glomeruli and tubules maintained their normal structure (Figure 3).

**Figure 4.**
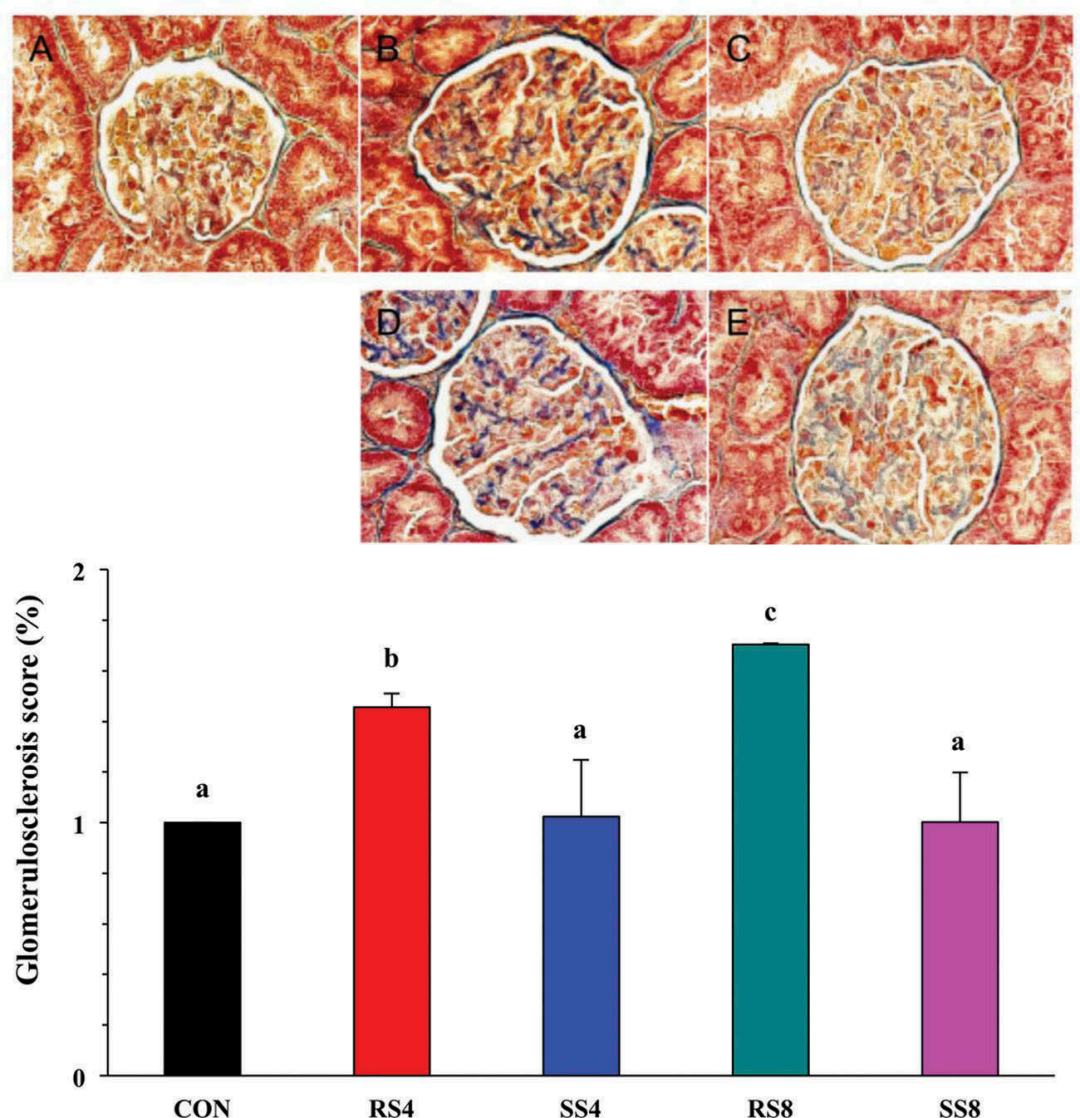
Effect of high-salt diet on kidney histopathology as revealed by Masson’s trichrome staining in the glomerular capsule and tufts (400× magnification). (a): CON, no fibrosis. (b): RS4, moderate fibrosis (arrows). (c): RS8, severe fibrosis (arrows). (d): SS4, and (e): SS8, very mild fibrosis (arrows). Graph on the bottom shows quantitative representation of renal injury in the kidneys of Dahl salt sensitive rats fed the different diets for 15 weeks. Glomerulosclerosis index for each rat was used to estimate the renal damage. Values are presented as mean ± SEM. Labelled means without a common letter differ significantly at *P *< 0.05.

To more quantitatively assess the extent of renal injury in the different salt groups, we performed Masson’s trichrome staining on the kidney sections (Figure 4, top) and calculated the glomerulosclerosis index scores from those (Figure 4, bottom). As expected, SS groups had significantly lower glomerulosclerosis scores than those of RS groups (*p* < 0.05). Within the RS groups, the RS8 rats showed higher glomerulosclerosis score than the RS4 rats (*p* < 0.05). This finding indicates that sea salt is much less damaging to the kidney than regular salt during high salt consumption.

### Blood chemistry

Blood chemistry results are shown in Table 4. In the case of plasma renin concentration, CON group was significantly higher than those of high salt diet groups (*p* < 0.001). Aldosterone levels followed the same pattern as renin wherein aldosterone concentration of CON group was higher than those of high salt diet groups. However, with both renin and aldosterone, there were no significant differences amongst the high salt diet groups.

**Table 4.** Biochemical indices of rats fed high-salt diets for 15 weeks.

	CON	RS4	SS4	RS8	SS8
**Plasma**					
Renin (mg/dL)	4.11 ± 1.13^b^	0.87 ± 0.15^a^	0.86 ± 0.33^a^	1.94 ± 0.45^a^	1.30 ± 0.26^a^
Aldosterone (pg/mL)	76.78 ± 31.52	21.74 ± 8.83^a^	16.15 ± 4.71^a^	24.58 ± 3.17^a^	16.33 ± 3.89^a^
**Serum**					
Na (mEq/L)	146.29 ± 0.94^a^	148.33 ± 0.62^a^	149.33 ± 0.61^b^	149.92 ± 0.83^b^	148.64 ± 0.70^b^
Cl (mEq/L)	101.14 ± 0.51^abc^	101.75 ± 0.37^bc^	102.50 ± 0.56^c^	100.83 ± 0.51^ab^	100.00 ± 0.60^a^
K (mEq/L)	5.97 ± 0.19^a^	6.75 ± 0.22^b^	6.73 ± 0.23^b^	6.61 ± 0.25^ab^	6.47 ± 0.24^ab^
Ca (mg/dL)	9.76 ± 0.11^a^	10.33 ± 0.13^b^	10.25 ± 0.10^b^	10.23 ± 0.12^b^	10.44 ± 0.12^b^
Mg (mg/dL)	2.41 ± 0.10^ab^	2.29 ± 0.03^a^	2.44 ± 0.07^ab^	2.35 ± 0.06^ab^	2.53 ± 0.06^b^
Osmolality (mOsm/kg)	308.43 ± 5.65^ab^	306.33 ± 5.85^a^	307.83 ± 3.66^a^	313.27 ± 4.63^bc^	315.25 ± 7.42^c^
**Urine**					
Aldosterone (pg/mL)	51.17 ± 4.75^a^	49.47 ± 2.36^a^	57.34 ± 3.01^a^	77.21 ± 3.91^b^	61.94 ± 6.58^a^
Na (mEq/L)	45.20 ± 6.41^a^	191.33 ± 19.59^c^	123.83 ± 13.78^b^	258.18 ± 22.40^d^	196.55 ± 22.55^c^
Cl (mEq/L)	66.80 ± 12.56^a^	202.17 ± 19.84^c^	129.17 ± 13.79^b^	276.73 ± 23.28^d^	232.36 ± 30.27^cd^
K (mEq/L)	78.47 ± 12.95^b^	49.51 ± 4.32^a^	57.83 ± 8.65^ab^	43.33 ± 8.68^a^	44.37 ± 5.21^a^
Ca (mg/dL)	9.78 ± 1.56^a^	25.77 ± 2.80^c^	17.23 ± 1.68^b^	20.17 ± 2.48^bc^	22.03 ± 2.18^bc^
Mg (mg/dL)	22.16 ± 4.44^a^	32.42 ± 4.37^ab^	32.77 ± 4.74^ab^	25.91 ± 5.71^ab^	38.27 ± 3.84^b^
Creatinine (mg/dL)	101.38 ± 21.06^ab^	97.81 ± 15.19^ab^	144.49 ± 21.22^b^	49.57 ± 9.94^a^	109.54 ± 20.04^b^
Osmolality (mOsm/kg)	931.50 ± 156.09	1022.67 ± 83.37	1140.17 ± 123.55	925.00 ± 130.61	1085.00 ± 111.24

Values are presented as mean ± SEM. Values with different superscripts within each row are significantly difference at *p* < 0.05 by Duncan’s multiple range test.

With regard to serum electrolytes, most of electrolytes were remarkably similar amongst all of the groups despite differences in salt intake. Both potassium and calcium concentrations of high salt diet groups tended to be higher than those of the CON group. Blood osmolality was similar amongst CON and the 4% salt groups, but tended to be higher in the 8% salt groups.

In general, urine analytes tended to be much more variable than those in serum. Urine aldosterone concentration in RS8 group was significantly higher than all other groups (*p* < 0.001). Not surprisingly, urinary sodium concentration of CON (45.20 ± 20.27 mEq/L) group was significantly lower than those of high salt diet groups (SS4: 123.83 ± 47.75 mEq/L, RS4: 191.33 ± 67.86 mEq/L, SS8: 196.55 ± 74.80 mEq/L, RS8: 258.18 ± 74.29 mEq/L), with the RS8 group being the highest (*p* < 0.001). Urinary chloride levels followed a similar tendency to urinary sodium with CON group at 66.8 mEq/L, and high salt diet groups ranging from 129 to 277 mEq/L (*p* < 0.001). Whereas potassium levels were higher in the CON compared to the salt groups, calcium and magnesium concentrations tended to be higher in high salt diet groups than in the CON group.

## Discussion

Continuous management through proper lifestyle choices and necessary medical treatment is recommended for the prevention of hypertension. The report of the Joint National Committee 7 [14] for lifestyle modification advocates five ways to control hypertension: diet therapy (dietary approaches to stop hypertension, DASH), weight reduction, sodium intake reduction, regular exercise and moderate alcohol consumption. According to a report in 2010, 1.65 million deaths from cardiovascular diseases among people in 66 countries (accounting for 74.1% of adult deaths throughout the world) were attributed to sodium intake above the recommended level of 2.0 g per day [15]. Although the mechanisms of salt-induced hypertension in the DSS rat are debated, one hypothesis by which NaCl consumption increases blood pressure is that as cellular sodium ions accumulate, cellular calcium concentration increases by Na⁺/Ca⁺ exchanger [16]. This rise in calcium results in the contraction of vascular smooth muscle and cardiac muscle, eventually leading to an increase in blood pressure. Interestingly, the anion accompanying sodium also plays an important role in determining the magnitude of blood pressure increase as DSS rats do not become hypertensive without chloride loading [17].

The main objective of our study was to explore the hypertension-inducing effects of dietary sea salt versus refined salt consumption using DSS rats fed chow containing either 4% or 8% of the two salts for 15 weeks. The ability to monitor and record precise blood pressure fluctuations in research animals is vital to research for human hypertension. Although direct, albeit invasive, measurement of blood pressure via implantable radiotelemetry devices is the preferred method over the traditional tail-cuff method, it is impractical for many groups due to its cost of acquiring and maintaining the necessary equipment as well as the need for surgical skills and training of its operator [18,19]. The tail-cuff method that we used was well suited for our study purposes and was performed by well-trained staff. While Dahl salt-resistant rats maintain normal blood pressure even when they are given a diet containing 8% salt [20], when DSS rats are given the same concentration of salt, both their SBP and DBP are increased [21]. These findings are in line with our results showing that SBP and DBP are increased in high salt diet groups. In our study, SBP of all groups were increased by 7%, 33%, 39%, 42%, and 58% between week 0 and week 15 for CON, SS4, RS4, SS8 and RS8, respectively. A similar trend was observed for DBP. The consistent differences in blood pressure between the RS4 and SS4 and between RS8 and SS8 are despite the sodium intake being similar. As shown in Table 1, although sea salt contains less sodium than refined salt, the variations in feed intake resulted in similar NaCl intake between RS and SS group rats. Thus, observed differences in the parameters between the RS and SS rats cannot be attributed to differences in sodium intake. All of these findings suggest that there are certain properties of sea salt that confers resistance to hypertension compared to refined salt.

How the sea salt diet actually exerts its anti-hypertensive effects is not clear. One obvious point to consider is the content of minerals other than sodium in the sea salt. On a weight basis sea salt that we used contains 85.7% NaCl whereas refined salt contained 99.9% NaCl. In addition to sodium, sea salt also contains calcium (1.5 mg/g), potassium (2.9 mg/g), magnesium (3.9 mg/g), and traces amounts of iron, manganese and zinc. It is known that dietary potassium can influence blood pressure, and that it can play a role in controlling blood pressure in hypertensive patients. Human subjects consuming high levels of potassium, for example, are less prone to hypertension even when the sodium intake is high [22]. In DSS rats, high potassium intake also attenuates increases in blood pressure caused by high sodium intake [23]. Two very recent high-profile studies point out that not only is potassium excretion inversely associated with systolic blood pressure for persons with hypertension compared to normotensive subjects [24], but that higher potassium excretion is associated with a lower risk of death as well as cardiovascular events [25].

Calcium intake has been associated with hypertension as well in that its intake enhances the relaxation of arterial vessel, thereby lowering blood pressure [26]. Relatively high intake of calcium has been linked to lower SBP in a population of Turkish elderly subjects [27]. Another important mineral for maintaining blood pressure is magnesium. A comprehensive and definitive study relating dietary magnesium and hypertension found that not only does blood pressure increase with magnesium deficiency but that the more severe the magnesium deficiency, the higher the blood pressure [28]. On the other hand, supplementing with 500–1000 mg/day of magnesium can lead to reduction in blood pressure by acting as a natural calcium channel blocker, thereby increasing nitric oxide and improving endothelial dysfunction [29].

To determine if sustained high blood pressure had an effect on heart function, we performed echocardiography on a subset of rats from all groups. Echocardiography plays an essential role in the evaluation of LV diastolic function, and the estimation of pulmonary hypertension pressures, the assessment of right ventricular size and function [30], especially thickening of the left ventricle, which is a key factor in hypertension [31]. In patients with hypertension, the ventricular wall becomes thick to maintain tension of left ventricular wall in proportion to chronic blood pressure load [32]. In our study, IVSs, IVSd, LVDd and LV mass values of RS4, SS8, and RS8 groups were significantly higher than those of the CON group, indicating abnormalities of the left ventricular function in our high salt groups. Interestingly, the SS4 rats were spared from these abnormalities despite comparable salt intake to the RS4 rats. These echocardiography results indicate that high salt diets generally lead to hypertrophy of the heart, and that sea salt consumption lessens this burden compared to refined salt intake.

In terms of organ weights, we found that heart and kidney weights of all the high salt diet groups were heavier than those of the CON group. In general, high salt intake leads to high water retention, perturbing the body’s system of homeostasis and in turn higher organ weight. These results are in agreement with the results of Aoi et al. [33] who reported that high salt diet induces increase in heart and kidney weight in DSS rats. With prolonged hypertension, damage to the kidneys is a common occurrence [34], and this is reflected in our observation of the high degree of glomerulosclerosis in the RS4 and RS8 rat kidneys (Figure 4).

The renin-aldosterone system (RAS) is critical in controlling sodium and potassium homeostasis, extracellular volume and arterial blood pressure [35,36]. In our study, plasma renin and aldosterone concentrations of RS4 and RS8 groups were higher than those of SS4 and SS8 groups, respectively. Furthermore, urinary aldosterone excretion was significantly higher in the RS8 group compared to the SS8 group. There data indicate that diets high in refined salt may cause higher blood pressure at least in part by disrupting the RAS homeostasis.

We conclude from our findings that both the level of salt intake as well as the type of salt can influence blood pressure. As expected, higher salt consumption led to higher blood pressure. However, even when the effects of salt concentration were ruled out, sea salt intake induced less hypertension than refined salt and caused less damage to the heart and the kidney. It is likely that the major beneficial effect of sea salt is associated with the mineral content of the sea salt that is known to be anti-hypertensive such as potassium, calcium and magnesium. It is also possible that there are as yet undetermined component(s) of the sea salt that might confer resistance to hypertension. Further studies are required to elucidate the mechanism of how sea salt attenuates blood pressure. Based on our findings it would also be important to determine if sea salt consumption would have similar effects on blood pressure in humans.
