# Corrigendum

**DOI:** 10.1080/16546628.2017.1300375

**Published:** 2017-03-20

Lee B-H, Yang A-R, Kim M-Y, McCurdy S and Boisvert W.A. (2017). Natural sea salt consumption confers protection against hypertension and kidney damage in Dahl salt-sensitive rats. Food & Nutrition Research. 61:1:1-10. http://dx.doi.org/10.1080/16546628.2017.1264713

When this article originally published online, the Acknowledgements section was incorrect. This has now been changed to read as follows:

This research was supported by the Globalization of Korean Foods R&D Program (funded by the Ministry of Agriculture, Food and Rural Affairs, Republic of Korea), and by the Chung-Ang University Research Grants in 2015.

